# Research Capacity Strengthening in Sub-Saharan Africa: Recognizing the Importance of Local Partnerships in Designing and Disseminating HIV Implementation Science to Reach the 90–90–90 Goals

**DOI:** 10.1007/s10461-019-02538-0

**Published:** 2019-05-16

**Authors:** Anna Kalbarczyk, Wendy Davis, Sam Kalibala, Scott Geibel, Aisha Yansaneh, Nina A. Martin, Ellen Weiss, Deanna Kerrigan, Yukari C. Manabe

**Affiliations:** 1 Johns Hopkins Bloomberg School of Public Health, 415 N Washington St, Baltimore, MD USA; 2 Department of Sociology, American University, Washington, DC USA; 3 Population Council, Washington, DC USA; 4 Office of HIV/AIDS, US Agency for International Development, Washington, DC USA; 5 Department of Medicine, Johns Hopkins University School of Medicine, Baltimore, MD USA

**Keywords:** HIV/AIDS, Capacity strengthening, Capacity building, Operations research, Implementation science

## Abstract

Capacity building in implementation science is integral to PEPFAR’s mission and to meeting the 90–90–90 goals. The USAID funded Project SOAR sponsored a 4 day workshop for investigators and governmental and non-governmental partners from 12 African countries. The workshop was designed to address both findings from a pre-workshop online needs assessment as well as capacity challenges across the capacity building pyramid, from individual skills to institutional systems and resources. Activities were output-oriented and skill based. An online survey evaluated sessions and changes in perceptions of needs; a majority of respondents strongly agreed that after the workshop, they better understood their personal and institutional capacity strengthening needs. Participants ‘strongly agreed’ that workshop content was relevant to their jobs (90%) and that they left the workshop with a specific plan for conducting future research (65%). Workshop results suggest that skill-building should be done in conjunction with systems capacity building within the cultural context.

## Introduction

Strengthening the skills and capacity of in-country individuals and institutions is an integral part of the President’s Emergency Plan for AIDS Relief (PEPFAR), the agency established by the United States (U.S.) government in January 2003 to address the growing global HIV epidemic. Strengthening capacity to conduct implementation science is a core tenant of PEPFAR’s HIV monitoring and evaluation framework [1] and a target of the 2030 Agenda for Sustainable Development [2]. Further, local capacity strengthening supports the Joint United Nations Programme on HIV/AIDS’ (UNAIDS’) 90–90–90 goal (90% of all people living with HIV will know their HIV status, 90% of all people with diagnosed HIV infection will receive sustained antiretroviral therapy and 90% of all people receiving antiretroviral therapy will be virally suppressed) [3]. The 90–90–90 targets have been widely adopted as a means of achieving control of the global HIV epidemic by 2030 [4–6]. Recently, the Office of Global AIDS Control which oversees PEPFAR, has begun funding local efforts to meet the 90–90–90 targets directly through USAID missions further underscoring the importance of regional capacity strengthening in implementation science.

Implementation science capacity strengthening involves working with in-country public health researchers, health care workers, and policy makers to ensure that data collection efforts and findings directly inform public health policies, strategies, and/or guidelines, which are essential for achieving the 90–90–90 goals and improving health outcomes more broadly [7]. The importance of implementation science in curbing the HIV/AIDS epidemic and the need to engage key actors such as local researchers and representatives of Ministries of Health in partnerships to this end, is well established [4–6]. A recent systematic review on the sustainability of health interventions in sub-Saharan Africa observes, however, a dearth of research on the methods and processes of ensuring intervention sustainability [8]. To date, capacity strengthening efforts in sub-Saharan Africa have had a more traditional focus on research skills such as data analysis or manuscript writing [9–15] leaving a critical gap in the regions and settings most impacted by the HIV epidemic for implementation science research capacity strengthening that acknowledges key multi-sectoral partnerships.

Project SOAR (Strengthening Operational AIDS Research) is a 5-year PEPFAR-funded cooperative agreement from the U.S. Agency for International Development (USAID) that assists local research institutions and individuals in the conduct and use of high-quality implementation science research to improve programs and policies and to ensure more efficient and effective delivery of critical HIV related services. Through 59 activities in 24 countries in sub-Saharan Africa (SSA), Project SOAR is identifying practical solutions to improve HIV prevention, care, and treatment services. To further strengthen and support implementation science research capacity, Project SOAR sponsored a 4 day workshop attended by 28 participants comprised of SOAR investigators and their governmental and non-governmental (NGO) partners representing 19 SOAR studies conducted in 12 African countries. The workshop was held in Johannesburg, South Africa, which serves as a central travel hub for regional work.

Given Project SOAR’s key emphasis on meeting in-country needs and research utilization, we sought to frame workshop content to emphasize the importance of building institutional and individual relationships with other stakeholders (including policymakers, programmers, and advocates) who are essential to the successful use of study results and the long-term sustainability of HIV research and programming. To our knowledge, this workshop is the first conducted in a sub-Saharan African context to focus on strengthening capacity in implementation science research skills and competencies within the context of institutional capacity. This paper describes the workshop’s theoretical foundation, agenda development and implementation, impact on participants, and implications and recommendations for future HIV implementation science capacity strengthening initiatives with the goal of informing the expansion of similar efforts.

## Methods

**Fig. 1:**
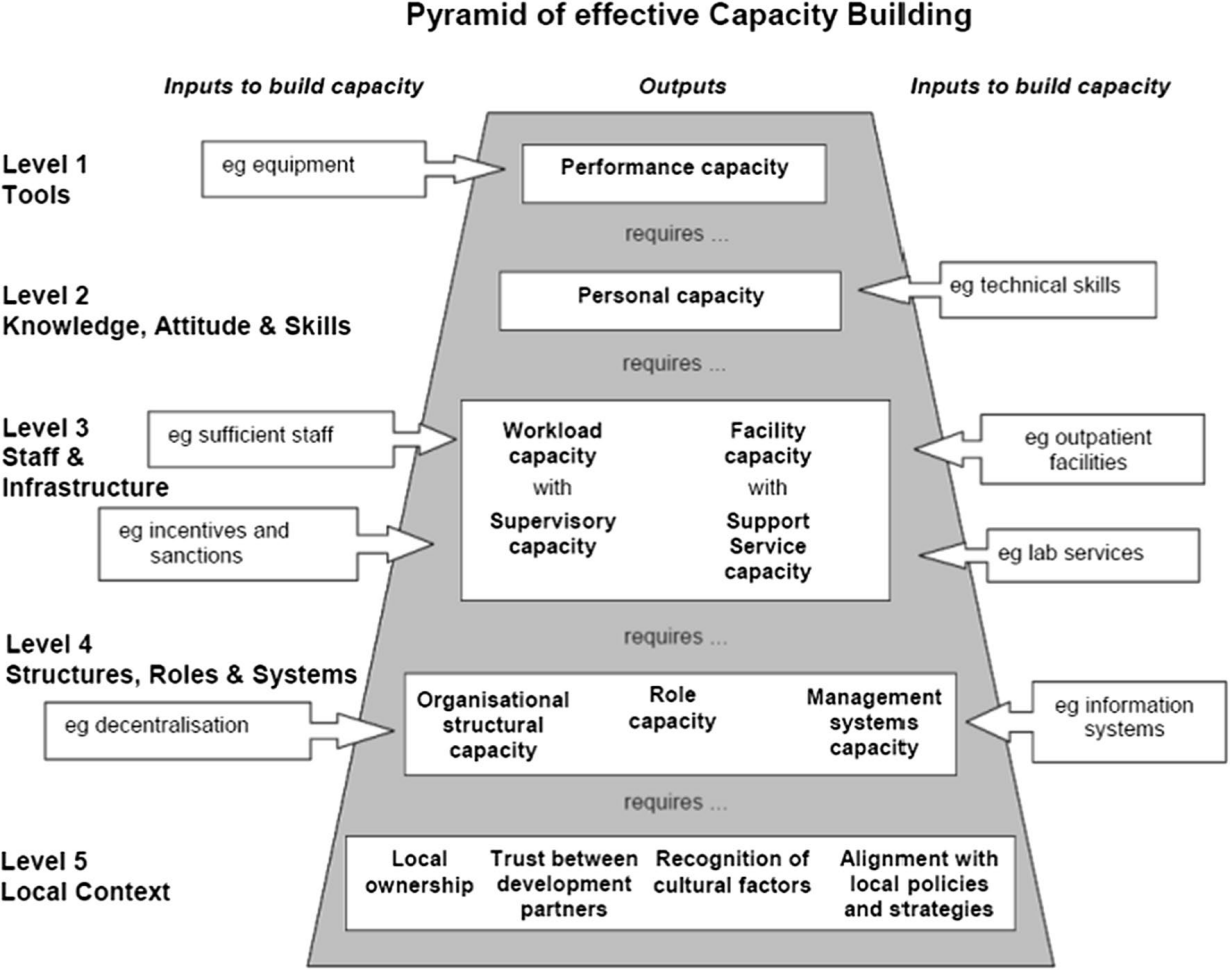
Capacity building pyramid

The “Capacity Building Pyramid” (see Fig. 1), first described by Potter and Brough [16] served as a conceptual model for the workshop design, content, and activities. The pyramid is a conceptual model which emphasizes institutional systems as essential for increasing the likelihood of sustainable capacity development regarding public health-related research [11]. Implementation science studies the efficient and impactful application of previous research findings towards practice, policy and scale-up. Understanding the health system and institutional capacity gaps is critically important to effective implementation; therefore, this pyramidal model is well-suited to implementation science research capacity strengthening as a framework.

Historically, capacity building workshops focus on the top of the pyramid and emphasize *individual* skill-building (e.g. data analysis and manuscript writing) [9–15]. For this workshop, we asked trainees to consider their organization’s *institutional* capacity—including their institution’s physical infrastructure, structures (e.g. record keeping and accountability), systems (e.g. flow of information and decisions), and roles (the lower levels of the pyramid)—as necessary for ensuring successful implementation of high-quality research. We also ensured that research utilization, networking, and cultural context were key areas of emphasis in the workshop agenda. Our team explored the feasibility and acceptability of using a workshop to focus on all levels of the Capacity Building Pyramid and assessed whether this approach yielded meaningful change in how participants identified and constructed personal and institutional research goals and agendas. The end goal was to encourage investigators to develop implementation science research questions that reflected the 90–90–90 goals, were locally and regionally important, were constructed in collaboration with governmental partners, and could ultimately be taken forward by the participants with mentorship provided by the SOAR consortium.

### Selection of Workshop Participants

Project SOAR principal investigators (PIs) engaged in ongoing operations research nominated African, locally-based colleagues for participation in the workshop based on the following three criteria: (1) the operations research study to which they were affiliated was in the process of collecting primary data or had an available data set to work with; (2) data from the study would make an impact on the field, locally or globally; and/or (3) members of the study team had a demonstrated need and interest in individual and institutional capacity strengthening. Nominated participants were involved in a range of HIV operations research projects which can be seen on the Project SOAR website (www.projsoar.org). Selected participants included researchers as well as key stakeholder partners from government (e.g. Ministries of Health and National AIDS Control Programs) and implementing program institutions with an equal proportion of governmental and non-governmental participants. Regional geographic balance was also sought to facilitate regionally-based small-groups. Workshop speakers and facilitators included experts from USAID, the Population Council, Johns Hopkins University, Palladium and partner institutions across SSA including the Infectious Diseases Institute (Uganda), University of Stellenbosch (South Africa), and the Perinatal HIV Research Unit of the University of Witswatersand (South Africa).

### Needs Assessment

Two months prior to the workshop, participants were asked to complete an online needs assessment in Qualtrics [17] an online survey platform, comprised of 37 questions. Questions were both multiple-choice and open-ended and divided into categorical blocks mapped to the pyramid (i.e. tools, skills, staff and infrastructure, etc.). Participants were also asked demographic questions and one open-ended question about their expectations for the workshop. The goal of the needs assessment was to understand individual and institutional needs and resources, individual learning priorities, and goals for the workshop. Data were exported to an Excel file, cleaned, and analyzed in Stata © version 12 [18].

### Curriculum Development, Activities, and Workshop Structure

The workshop curriculum was designed based on the results of the needs assessment in conjunction with internal Project SOAR leadership conversations. Talks and activities were designed to be output-oriented, focusing on one of the four themes that emerged from the needs assessment: institutional needs and resources, supplemental research questions based on SOAR studies, dissemination and utilization strategies, and network development. The development of these deliverables through different activities is described in additional detail below. Institutional needs and resources referred to self-identified gaps and strengths of their institution reported by each participant and characterized the diverse research and practice environments.

The deliverable for the first part of the workshop was to reflect on the organizational infrastructure, institutional needs, and resources needed to efficiently and effectively conduct implementation science research in addition to individual skills obtained in workshops. The second deliverable was supplemental research questions identified by workshop organizers and participants jointly as a priority skill for development of research and grant writing capacities in small groups. Participants were then instructed to examine the parent study’s primary research questions and develop secondary aims that could further enrich the study or lead to additional operations research. Dissemination and utilization strategies included strengthening of interpersonal communication skills via constructing and delivering short 30 second elevator pitches, understanding audiences, and effective strategies for engaging multiple stakeholders in research dissemination. Finally, network development activities were designed to introduce participants to existing professional networks and tangible skills for developing informal and formal networks of their own.

Given the ‘output-oriented’ goals of the workshop, significant time was allotted each day for strategically constituted small groups to work together with facilitators to produce and present materials. Workshop activities were developed with the intention of fostering partnerships between researchers and policymakers on Project SOAR studies wherever possible. Small groups were organized according to the activities of that day, e.g. pairing researchers and policy makers or program implementers when discussing how to engage stakeholders or grouping participants in the same institution type and field when discussing institutional needs and resources. Facilitators and presenters were identified based on areas of implementation science expertise; emphasis was placed on identifying presenters from sub-Saharan Africa.

The project leadership team took detailed notes during each talk, activity, and small-group session. Debriefing sessions were also held at the end of each day which allowed the team to reflect on the themes and outputs. Notes from these activities were later compiled and reviewed manually by two members of the study team for accuracy and emerging themes.

### Post-workshop Survey

The post workshop evaluation was developed in Qualtrics© and disseminated via email to all participants 2 weeks after the workshop. Participants were asked to answer 26 demographic, Likert scale, and open-ended questions about the value of each presentation and small group session to them both personally and professionally within the context of their current institution. The evaluation included questions about how likely participants were to use the skills covered and resources offered in the training and to engage with Project SOAR in the future including via networks and a small grant initiative to be introduced by SOAR management.

## Results

### Needs Assessment

Respondents represented a wide range of stakeholders from project coordinators to government officials. Twenty-six of the twenty-eight (92.8%) participants responded to the needs-assessment. The majority of participants were either affiliated with an NGO (52%) or a government agency (44%), while a minority worked for a university (4%).

Seventeen (65%) respondents had attended a workshop or short-course in the past 2 years with the majority participating in training that had been focused on data analysis (10/17) and study design (8/17). Many participants said they had access to or had in place data management and analysis systems (88%), core lab support (39%), grant management systems (70%), audit mechanisms (86%), and strategic plans (83%). 42% also reported having an identified in-country mentor, though fewer (23%) reported having an identified international mentor.

Respondents were asked to rank their priorities for learning or improving skills on a scale from 1 to 10 with one being the highest priority and 10 being the lowest. Respondents ranked data analysis (3.45) and study design highest (5.2). Grant writing (7.2), report writing (7.25), and interpreting or utilizing research (7.3) were among the lowest ranked priorities. The majority of respondents ranked data collection, research protocol writing and implementation, and presenting to a local working group as skills they felt capable of performing. Overall, there was low reported capacity in data analysis, data collection, and grant writing, paradoxically among those who had reported previously taking courses on those subjects. Additional data on reported capacities and prior training activities are presented in Table 1. The supplementary research question development was designed to address the expressed priority need in study design and low reported capacities in grant writing.

**Table 1:** Reported capacity by training courses taken

Reported capacities	Prior training activities						
	Quant. data analysis	Qualitative data analysis	Cost effectiveness analysis	Study design	Study tool design	Developing dissemination strategies	Writing reports
	(n = 8)	(n = 7)	(n = 2)	(n = 9)	(n = 7)	(n = 4)	(n = 6)
Write a research protocol	5 (63)	5 (71)	1 (50)	5 (56)	4 (57)	1 (25)	2 (33)
Implement a research protocol	8 (100)	7 (100)	2 (100)	8 (89)	7 (100)	4 (100)	5 (83)
Collect data	8 (100)	7 (100)	2 (100)	8 (89)	7 (100)	4 (100)	5 (83)
Analyze data	3 (38)	3 (43)	1 (50)	4 (44)	2 (29)	1 (25)	1 (17)
Give a presentation at a large conference	2 (25)	2 (29)	0 (0)	4 (44)	2 (29)	2 (50)	3 (50)
Give a presentation at local working group meeting	7 (88)	6 (86)	1 (50)	9 (100)	7 (100)	4 (100)	6 (100)
Write a first author paper	4 (50)	3 (43)	2 (100)	4 (44)	2 (29)	2 (50)	3 (50)
Write a grant proposal	1 (13)	1 (14)	0 (0)	2 (22)	1 (14)	2 (50)	2 (22)
Develop a budget and budget justification	7 (88)	6 (86)	2 (100)	6 (67)	7 (100)	3 (75)	4 (67)
Run a stakeholder meeting with policy makers	4 (50)	5 (71)	1 (50)	5 (56)	4 (57)	3 (75)	5 (83)
Disseminate results	3 (38)	4 (57)	2 (100)	3 (33)	2 (29)	3 (75)	3 (50)
Total	8	7	2	9	7	4	6

### Workshop Implementation

As noted above, the workshop curriculum was designed around four themes that emerged from results of the needs assessment and internal leadership conversations.

Theme 1—Identifying institutional needs and resources. After a framing talk (Tom Kakaire, Infectious Diseases Institute, Makerere College of Health Sciences) about the capacity building pyramid [14], small groups were organized according to organization type (government, NGO, research) and asked to identify personal and institutional needs at each level in the pyramid and present an organized summary of their needs. Government workshop participants highlighted the need for government workers to gain stronger research skills so that they could meaningfully assist research and NGO partners both in setting priorities as well as in research program implementation. Government representatives also highlighted the importance of linking government and research agendas. They noted the importance of engagement from the research side “early and often” to ensure regular policy review against the national agenda and harmonization of research efforts to focus on national priorities.

Program and academic workshop participants highlighted the importance of data collection skills for their staff, especially in rural areas, and research skills for themselves including data collection, data analysis and writing up data. They also emphasized structural deficits such as the need for better research facilities, and for facility-based expertise in data management and grants management and financial post-award management.

Workshop participants from each group suggested a lack of personal agency in resolving resource needs and institutional barriers to high quality research led by Africans. However, workshop facilitators highlighted the importance of identifying the components that lead to better internal systems noting that breaking systems down into manageable pieces provides a path for achievable, incremental change.

Theme 2—Designing supplemental research questions. Expert researchers both from SSA and from the U.S. began this session with a discussion around building supplemental research questions that could be answerable from the existing data for the study’s primary research questions, or with minimal additional data collection. Small groups were organized by research themes (HIV Prevention Strategies, HIV Treatment, Care and Support, Key Populations at Risk for HIV, Prevention of Mother-to-child Transmission of HIV) with governmental and NGO participants placed together in topic area groups. At the end of this session, small groups presented their supplemental questions to workshop participants and Project SOAR leadership to receive feedback. Participants noted that supplemental questions can serve as a valuable tool to respond to issues that arise during the study in real-time, and several requested clarifications about logistics and potential study populations that could answer gaps in the primary research questions. Participants were then tasked to refine their supplemental questions based on feedback from workshop participants and facilitators. Of note, soon after the workshop, two participants submitted supplemental research questions developed at the workshop and successfully received funding through a regional small grants program.

Theme 3—Dissemination and utilization strategies. This session began with a short talk on how to make an ‘elevator speech’ about a research project or future proposal. In a role-playing exercise, participants were asked to describe and pitch their study as if to a minister of health or a sponsor. Participants in the ‘audience’ were asked to comment on the effectiveness of the pitch and suggest strengths and weaknesses at the end of each role play. Participants left this session recognizing the usefulness of preparing mental notes or speeches in advance and of being confident in their delivery as well as of the importance of building personal relationships in short interactions. They also left with an appreciation of the concept of “beginning with the end in mind” [19]. Workshop presenters stressed that involving policy makers early in the discussion of research questions and involving them throughout the conduct of research and at the time of dissemination increases the likelihood of uptake. One participant observed that involving the parliament allowed them to provide research findings for the development of national evidence-based policy.

Theme 4—Network development. The importance of networking as a way to multiply the impact of research was discussed and networks were described as a way to foster mentorship both at home and abroad. The keynote speaker, Marietjie DeVilliers, from the University of Stellenbosch highlighted the importance of building personal relationships as a foundation of network development. Dr. DeVilliers reviewed existing networks and introduced AFREHealth (https://www.afrehealth.org/), a new interdisciplinary health professional forum. In follow up discussions, small groups, organized by region, discussed how to develop a network for their geographical or technical areas. Participants asked questions about how to engage alumni from other networks, and how to leverage networks efficiently to retain local capacity and mitigate the loss of personnel to higher-income settings. Facilitators and participants commented that building networks takes time, leadership, and a shared desire.

### Evaluation

Twenty participants (20/28 or 71.4%) responded to the post work-shop survey. As shown in Table 2, the majority of respondents strongly agreed that after the workshop, they felt differently about both their personal (85%) and institutional (60%) capacity strengthening needs and were more aware of the lower levels of the Capacity Building pyramid including “Structures, Roles & Systems” and “Local Context”. This was particularly true for participants from government agencies. In the evaluation, the individual capacities ranked most important were grant writing (89%), research utilization (85%), and research interpretation (85%). The majority of respondents (75%) said that, after, the workshop, they strongly agreed that they felt more comfortable engaging with someone from a different profession (i.e. researchers reported improved capacity engaging with government officials and vice versa).

**Table 2:**
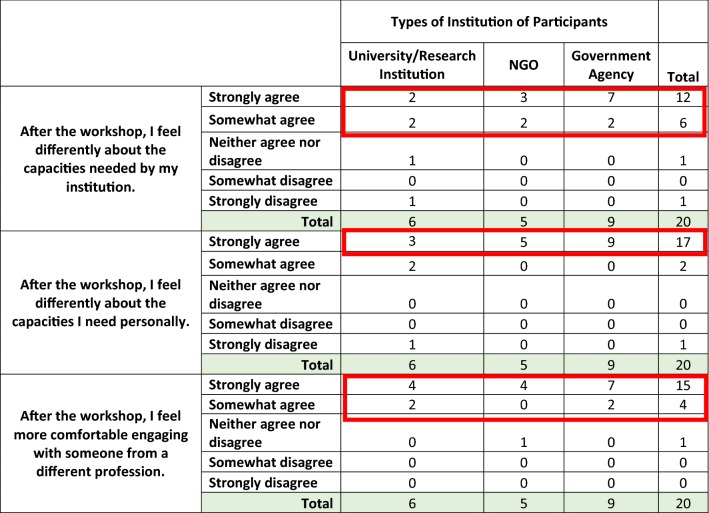
Changes in perceptions of capacities after the workshop by participant institution type

Participants overwhelmingly ‘strongly agreed’ (90%) that the workshop content was relevant to their jobs. When asked if presentations and small group sessions were valuable to individual learning and growth, the majority of respondents ranked each presentation (between 60 and 90%) and small group session (between 55 and 80%) as very valuable. Giving an elevator pitch was the most commonly reported skill strengthened by the workshop. Many respondents (65%) strongly agreed that after the workshop they left with a specific plan for future research they would like to conduct or support as part of an outgrowth of their Project SOAR study. Respondents were also asked to identify which tasks they completed during the workshop: 21% said they networked with someone new, 18% developed a research questions, 18% identified professional needs for themselves, and 15% developed a dissemination and utilization plan. Responses to open-ended questions were generally positive but lacked specificity.

## Discussion

In this implementation science research capacity strengthening workshop, activities were structured around the Capacity Building Pyramid [11, 16]. Activities at the bottom of the pyramid such as Local Context (level 5), Structures, Roles and Systems (level 4) and Staff and Infrastructure (level 3), which are central tenets of successful implementation research, were given equal emphasis to activities at the top of the pyramid including Tools (level 1) and Knowledge, Attitude and Skills (level 2) in contrast to traditional research capacity strengthening which tends to emphasize activities at the top of the pyramid. This reflects a growing appreciation that the 90–90–90 goals cannot be reached without strong implementation science research and research capacity strengthening that appreciates and addresses local context and engages local stakeholders.

While participants did not strongly identify needs in the bottom levels of the pyramid in the pre-workshop assessment, through the course of the workshop participants developed an appreciation of the critical importance of these components in successful research utilization and sustainability. They came to understand their own and their institutions’ needs in these domains and embraced and appreciated the activities that focused on these lower levels. For example, participants were able to identify their own skill deficiencies in engaging local stakeholders to make research relevant to the local context. This, in turn, led to keen participation in sessions such as the elevator speech presentation and exercise that offered skills in explaining to a stakeholder, not only the final study findings, but the purpose of the study prior to finalization giving stakeholders the opportunity to anticipate findings. This approach fills an important gap in current capacity strengthening efforts that concentrate on building individual skills, but do not necessarily consider the critical need for a strong implementation science skills set and the central importance of organizational infrastructure and context in successfully pursuing and conducting locally relevant operations research grants.

The results of our workshop show that skill building at the top of the pyramid (basic skills and tools training) remains important but needs to be done in parallel to the kinds of implementation science skill building found at the bottom of the pyramid. Workshops should be output-oriented, providing participants with tangible products they can use in the future. In this workshop, outputs included refined supplemental research questions and an elevator speech. Workshops should also have a strong focus on the strategic selection of participants that facilitates stakeholder engagement and should focus on contextually driven research with key local stakeholder engagement and buy-in (i.e. bottom of the pyramid). Many workshop participants came from strong indigenous research institutions. For them, learning to engage governmental stakeholders early in the planning phases of research protocol development and throughout data collection and analysis ensures ownership over the conclusions emphasized during final dissemination as well as in their utilization and sustainability.

There were a few limitations to the implementation of this workshop. The sample of participants was limited to the SOAR consortium, which, while geographically diverse and highly representative of different groups (i.e. 50% government) cannot be considered representative of the larger cohort of mid-career Africans working in the field. The workshop was also relatively short, at 4 days. The need to link participants with mentors and follow-up support became apparent during the workshop given the relatively low proportion of participants who were able to complete the ‘deliverables of the workshop. Ongoing mentorship will be needed to advance and ensure the successful achievement of the goals and ideas generated in the workshop. There were also no specific additional resources to build institutional capacity beyond the training itself, a key area of focus of the workshop content. Open-ended questions in the survey materials were rarely addressed by respondents and did not reveal additional information or clear areas for improvement.

This workshop was the first piece of an ongoing effort to provide implementation science capacity strengthening to local Project SOAR investigators. At the conclusion of the workshop, Project SOAR leadership introduced a small grants program that includes mentorship and networking opportunities and that may provide a mechanism for funding certain projects developed in the workshop. The small grants program encourages mid-level SOAR investigators to pursue implementation science questions such as secondary research questions, additional research utilization opportunities, and knowledge translation activities. A follow-up workshop is also being planned and will include small grant awardees and their mentors. This second workshop will focus on strengthening proposals, mentorship and the development of a mentorship plan, and fostering collaborative relationships between small grant awardees and local partners in the areas of implementation science and mentorship. These activities support not only Project SOAR’s overarching aim to improve local HIV prevention research capacity, but also strengthen participating investigator’s methodological and practical skills to help reach the 90–90–90 goals in sub-Saharan Africa.
